# Accuracy of 3D Tooth Movements in the Fabrication of Manual Setup Models for Aligner Therapy

**DOI:** 10.3390/ma15113853

**Published:** 2022-05-28

**Authors:** Hisham Sabbagh, Sebastian Marcus Heger, Thomas Stocker, Uwe Baumert, Andrea Wichelhaus, Lea Hoffmann

**Affiliations:** 1 Department of Orthodontics and Dentofacial Orthopedics, University Hospital, LMU Munich, Goethestrasse 70, 80336 Munich, Germany; hisham.sabbagh@med.uni-meunchen.de (H.S.); thomas.stocker@med.uni-meunchen.de (T.S.); uwe.baumert@med.uni-muenchen.de (U.B.); andrea.wichelhaus@med.uni-meunchen.de (A.W.); 2 Private Practice “Dr. Heger”, Fürther Strasse 2, 90429 Nürnberg, Germany; info@zahnarzt-drheger.de

**Keywords:** clear aligner, aligner, orthodontic appliance, dental casts

## Abstract

Background: The clinical outcome of aligner therapy is closely related to the precision of its setup, which can be manually or digitally fabricated. The aim of the study is to investigate the suitability of manual setups made for aligner therapy in terms of the precision of tooth movements. Methods: Six dental technicians were instructed to adjust each of eleven duplicate plaster casts of a patient models as follows: a 1 mm pure vestibular translation of tooth 11 and a 15° pure mesial rotation of tooth 23. The processed setup models were 3D scanned and matched with the reference model. The one-sample Wilcoxon signed-rank test (*p* < 0.05) was used for evaluation. Results: The overall precision of the translational movement covers a wide range of values from 0.25 to 2.26 mm (median: 1.09 mm). The target value for the rotation of tooth 23 was achieved with a median rotation of 9.76° in the apical-occlusal direction. Unwanted movements in the other planes also accompanied the rotation. Conclusions: A manual setup can only be fabricated with limited precision. Besides the very high variability between technicians, additional unwanted movements in other spatial planes occurred. Manually fabricated setups should not be favored for aligner therapy due to limited precision.

## 1. Introduction

Treatment with aligners is an integral part of orthodontic therapy. Aligners are mainly used for the treatment of moderate crowding and spacing [1,2,3,4], for protrusion and retrusion, and minor intrusion and extrusion movements of teeth [4,5,6]. The therapeutic outcome closely correlates with the type and direction of the planned tooth movements. Tooth movements are achieved in increments, starting from the initial malocclusion to achieve the final setup [3,7]. The number of intermediate steps depends on the system used and the extent and type of tooth movements, e.g., Invisalign^®^ (Align Technology, San Jose, CA, USA) usually employs movement increments of 0.25–0.33 mm [8], CA^®^ (Scheu-Dental, Iserlohn, Germany) 0.5–1 mm [9], Essix^®^ (Dentsply, Charlotte, NC, USA) 1 mm [10], and ClearSmile^®^ 0.5 mm [11]. A corresponding number of setup models is therefore required for the fabrication of the aligners. Setup models can be created manually or with the aid of a CAD/CAM system. Today, the manual fabrication of aligner setups on plaster casts is increasingly being replaced by digital setups and 3D-printed models [12]. However, manual setup fabrication remains a common method for in-house aligner fabrication, as the necessary hardware (scanners and 3D printers) and software are not ubiquitously available.

The accuracy of setup models is essential owing to the small movement increments in aligner systems. The inaccurate implementation of tooth movements in the setup is a possible reason for the difference between setup and patient outcome [13]. An investigation comparing digital and manual setups according to the ABO objective grading system (ABO OGS) showed only small differences [14]. However, the ABO OGS does not take into account the precision of each individual movement step’s implementation. While translational and rotational tooth movements can be precisely executed in a digital setup (with 1/10 mm and/or 1/10° precision, depending on the software used), the precision and suitability of manual setups for aligner therapy has not yet been investigated. Therefore, the aim of this study was to investigate the precision of a defined translational tooth movement and a defined rotational tooth movement in a manual setup.

## 2. Materials and Methods

A plaster cast of an upper jaw with anterior crowding served as the reference model, from which a total of 72 identical casts were duplicated. Six dental technicians (A–F) experienced in the fabrication of manual setups agreed to participate in this study. Participating technicians had at least five years of experience and received training in the form of a hands-on course prior to the start of the study to ensure consistency. Each of them was instructed as follows: from the occlusal aspect, tooth 11 should be moved purely translationally 1 mm in the vestibular direction and tooth 23 should be rotated 15° in the mesial direction. To avoid familiarization effects, the technicians were instructed to process only one model per day. The work was to be completed within three months. Each technician was provided with standardized working instructions and twelve casts, of which eleven should be processed by the technician. The twelfth served as an unchanged reference cast. All setup casts were provided with vestibular and palatal silicone keys (Tresident 2000K, Schütz dental GmbH, Roßbach, Germany). The silicone keys served to reproduce the initial positions of teeth 11 and 23 at any time. An axial marking on the teeth being moved served as a rotation and reset guide.

After completion, the reference casts and processed setup models were digitized using a desktop scanner (KaVo Everest, KaVo Dental GmbH, Biberach/Riß, Germany). The scans were reviewed for scan artifacts (Everest Scan Control program, KaVo Dental GmbH), corrected if necessary, and then saved as STL files. From each STL file, teeth 11 and 23 were isolated using the program GOM Inspect (GOM GmbH, Braunschweig, Germany) and individually saved as STL files. Thus, virtual models of the entire dental arch and of the two exposed teeth were available for a software-assisted analysis using MeshLab v.1.3.2 (CNR-ISTI; Pisa, Italy) [15].

**Figure 1:**
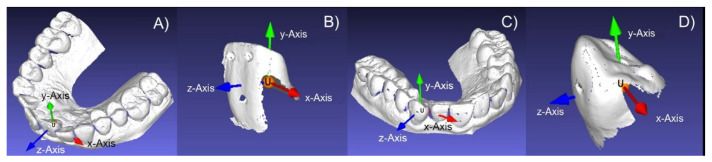
Orientation of the coordinate systems relative to the crown: tooth 11 in the dental arch (**A**) and the separated tooth (**B**); tooth 23 in the dental arch (**C**) and the separated tooth (**D**).

Using MeshLab, a coordinate system was placed in each STL file. Its system’s origin (*x* = 0, *y* = 0, *z* = 0) was positioned in the crown of teeth 11 or 23, respectively (Figure 1), in such a way that its *x*-axis pointed in the mesio-distal direction, its *y*-axis in the apical-occlusal direction, and its *z*-axis in the orovestibular direction.

**Figure 2:**
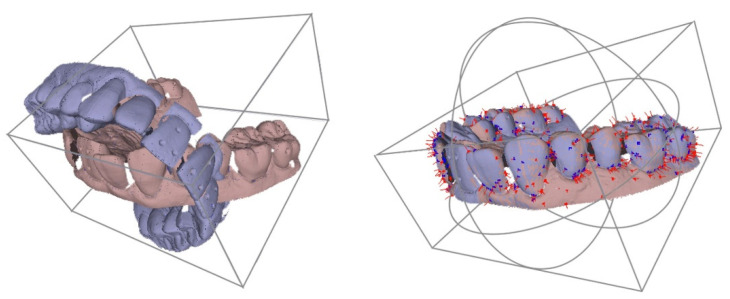
Software-based superimposition of comparison model (orange) and setup model (blue) using MeshLab’s “Align Tool” and reference points.

Afterwards, the scans of a processed model and the initial situation of each setup were congruently superimposed using an iterative closest point algorithm (ICP) as implemented in MeshLab’s “Align Tool” [15] with the following settings: 1000 samples, target distance was set to “0”, and 100 iterations were carried out (Figure 2).

**Figure 3:**
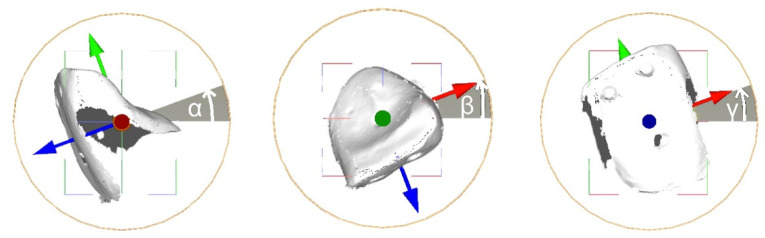
Tooth movement calculation using MeshLab’s roto-translation matrix. The rotation angle was calculated with a rotation matrix according to “the roll, pitch and yaw convention” (accuracy ± 0.2 mm).

In order to use this algorithm, an initial approximate alignment of the scans was necessary, which was acquired by matching the single coordinate systems of both scans (Figure 3).

Based on the inaccuracies of the scanner (±20 µm, according to the manufacturer) and potential errors arising during the superimposition, we estimated the accuracy of the calculated values to be ±0.2 mm.

Descriptive and inferential statistical analyses were conducted using IBM SPSS Statistics 25 (IBM Corp., Armonk, NY, USA). For each combination of tooth, axis of movement, and direction of movement, descriptive statistics were reported as median and interquartile range (IQR). Additionally, mean, standard deviation, range, median, and IQR were tabulated. Due to the sample size (*n* = 66) and violation of the assumption of a normal distribution, a one-sample Wilcoxon signed-rank test (*p* < 0.05) was used to test whether the median of the samples was equal to the target values (Table 1). Post hoc power analysis was performed using the absolute values of means and standard deviations and a two-tailed Wilcoxon signed-rank test (one-sample case) with α = 0.05 and *n* = 66 (G*Power version 3.1.9.6 for Mac) [16]. For rotational movements, the absolute mean difference between prescribed and actual rotation was 5.49 ± 3.28°, and for linear movements, the absolute mean difference was 0.34 ± 0.25 mm. In both cases, the achieved power was >0.99.

## 3. Results

### 3.1. Orovestibular Translation of Tooth 11, 1 mm Vestibular

**Figure 4:**
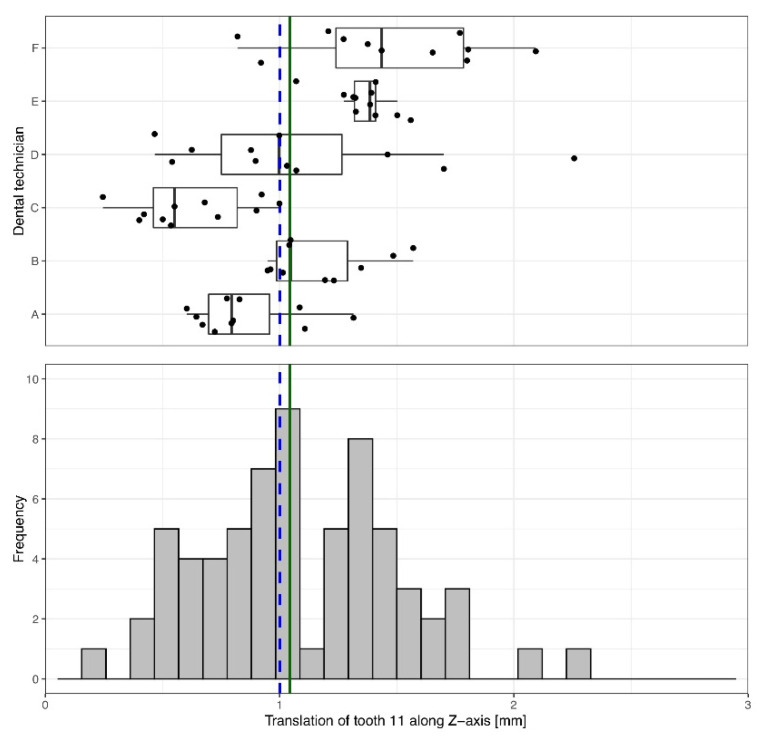
Determined orovestibular (*z*-axis) translational movement of tooth 11. A movement of 1 mm (blue dashed line) was specified. Across all technicians, a median movement of 1.04 mm (green solid line) was achieved. The box-and-whisker plot in the upper panel shows the measured movements of each individual dental technician. The histogram in the lower panel depicts the overall distribution of the measurements.

The overall measured precision of the translational movement covers a very wide range of values from 0.25 mm to 2.26 mm (Table 1). However, the median (and 95% interquartile range) translational movement of tooth 11 was 1.04 mm [0.79 mm; 1.39 mm] in the orovestibular direction, which was close to the proposed value of 1 mm (Z = 1.434; *p* = 0.152) (Table 1, Figure 4).

In addition to the proposed orovestibular translational movement, tooth 11 was also moved 0.18 mm [0.09 mm; 0.28 mm] mesio-distally (*x*-axis) and 0.13 mm [−0.33 mm; 0.02 mm] vertically (*y*-axis) (Table 1). Both unintentional movements were significantly different (*p* < 0.001) from the proposed value (0 mm) and showed a wide range of variation: in the mesio-distal direction between 0.01 mm and 0.58 mm and along the vertical axis between 1.02 mm and 0.37 mm (Table 1).

Additionally, unwanted rotations were introduced into the setup (Table 1). The median rotation around the *x*-axis was 2.62° [1.77; 4.35], around the *y*-axis −0.91° [2.15°; 0.05°], and around the *z*-axis 1.38° [2.39°; −0.81°]. All unintentional rotations were different in statistical significance (*p* < 0.001) from the proposed rotation.

Inter- and intra-technician comparisons revealed large deviations (Figure 4). Some technicians worked with good precision but less trueness. Others worked with good trueness but less precision. While dental technician E exhibited only a slight variation in the proposed setup, dental technician D showed a wider range of variation in the setup.

### 3.2. Mesio-Rotation Tooth 23, 15°

**Figure 5:**
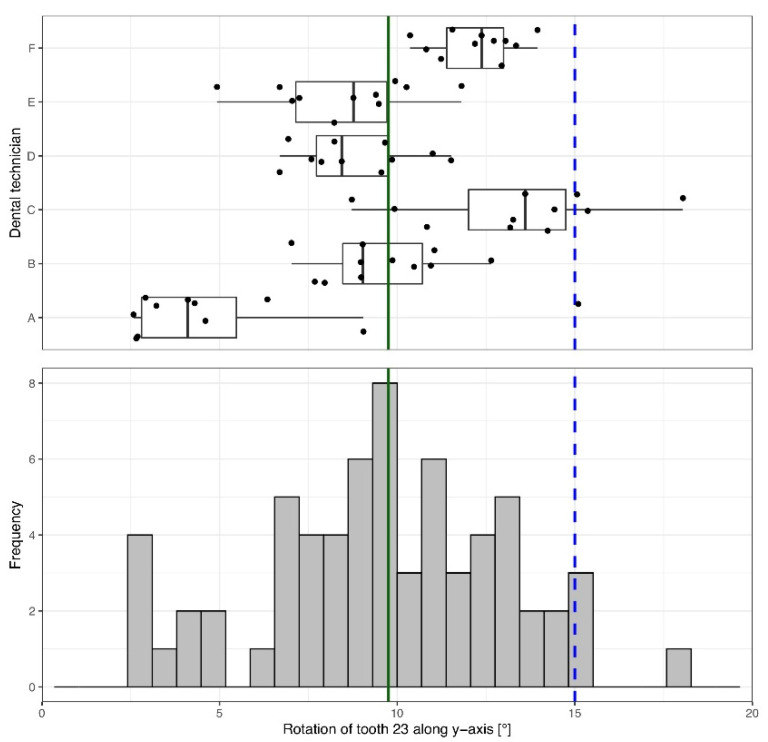
Rotation of tooth 23 around the *y*-axis (apical-occlusal). A rotation of 15° in the mesial direction was specified (blue dashed line); the median achieved across all technicians was 9.76° (green solid line). The box-and-whisker plot in the upper panel shows the measured movements of each individual dental technician. The histogram in the lower panel depicts the overall distribution of the measurements.

A median vertical rotational movement around the *y*-axis of 9.76° [7.58°; 12.19°] was applied to tooth 23 (Table 1, Figure 5).

Although only a 15° mesial rotational movement was requested, additional rotational and translational movements of the crown along the *x*-, *y*- and *z*-axes were applied (Table 1). Tooth 23 was moved −0.26 mm [−0.37 mm; −0.14 mm] along the *x*-axis (mesio-distal), 0.14 mm [0.02 mm; 0.33 mm] vertically along the *y*-axis, and −1.01 mm [1.16 mm; −0.75 mm] along the *z*-axis (orovestibular). Most movements significantly deviated (*p* < 0.001) from the proposed setup (Table 1). Additional rotational movements along the *x*-axis (−0.52° [−3.57°; 1.70°]; Z = −1.607, *p* = 0.108) and the *z*-axis (4.47° [2.99°; 7.02°]; Z = 7.056; *p* < 0.001) also occurred.

Results showed a wide intra- and inter-technician variation of the derotation accomplished (Figure 5). Some technicians worked with precision but less trueness. Others worked with high trueness but less precision. Only two of the dental technicians (A, C) achieved the proposed mesial derotation of 15° for tooth 23 (Figure 5). On average, the tooth was derotated less than specified.

## 4. Discussion

The results of this study illustrate the different perceptions of each individual dental technician concerning the position of a tooth and the requested movement in the setup. Although the exact final positions of each tooth were defined by the practitioner, the implemented movements of the teeth showed a very high inter- and intra-technician variability.

The consideration of six experienced operators in this study may not be representative of dental technicians as a whole; however, the implementation of planned tooth movements during manual fabrication of aligner setups does not appear to be possible with sufficient precision and reproducibility, as the specified simple tooth movements were not achieved even under study conditions involving training and detailed instructions.

In aligner therapy, the precision of tooth movements in the setup is of utmost importance, as the fit between the aligner and the tooth surface affects the transmission of orthodontic force and the onset of tooth movement [17,18,19]. Inaccuracies in the setup must be considered in addition to other critically discussed inaccuracies in aligner fabrication, such as model manufacturing [20,21] and thermoforming [22]. In 3D-printed models for aligner therapy, deviations of less than 0.25 mm compared to the virtual models are necessary [23]. After thermoforming, the resulting gap width between tooth surfaces and aligners was found to range between 0.10 mm and 0.35 mm, depending on the intraoral region and aligner material [19]. Since deviations as small as 0.10 mm may be sufficient to affect the predictability of tooth movement [17], the introduction of additional variability through manual setups seems impossible. Rather, variations due to aligner fabrication should be reflected and accounted for in the setup, for example, by adjusting movement staging in specific regions [17].

Nevertheless, the precision of the individual tooth movements should not be confounded with the overall quality of the setup. While individual tooth movements (translation, rotation) can be precisely and reproducibly executed in digital setups [24], the overall quality of the setup depends on the total tooth movements performed and relies on the operator for both manual and digital setup fabrication. For example, significant differences in the ABO OGS scores between two digital setups of the same original models made by one clinician were found [25].

The precision of the tooth movements is also relevant with regard to the forces occurring during aligner therapy. Experimental studies show that, even with small movement steps in the setup, forces of varying degrees occur. These forces vary depending on the individual malocclusion, step size, material, and aligner extension [26,27]. It may therefore be assumed, that the deviations from the desired movement described in this study are likely to result in undesirable or uncontrolled forces and moments due to the imprecision of the manual setup. The rotation of tooth 23 (15° rotation around *y*-axis) was accomplished with a median rotation of 9.76° (range: 2.58° to 18.04°). The results for the translational, orovestibular movement of tooth 11 show a wide range of 0.25–2.26 mm (i.e., a difference of 2.01 mm), which is also not acceptable. It is out of the question that smaller steps are clinically advisable and more effective in aligner therapy [28,29]. In order to reflect the maximum range of translational tooth movement within the manual setup, this study applied a target step of 1 mm, which is within the range given by some manufacturers [30]. A similar approach was used for the rotational movement [31]. We expect that implementing smaller, more difficult-to-control movements would result in an even lower precision in the manual setup process. Such sizeable deviations in the setup model can lead to higher forces [26] or a retardation of tooth movement [32,33]. Excessive forces during orthodontic tooth movement may in turn induce external resorption [34,35,36,37].

According to our results, a manual setup can only guarantee a limited predictability of clinical tooth movement. Our study shows that it is difficult to manually move a tooth along one axis without simultaneously inducing movements along the other axes. The non-prescribed movements even exceeded a usual clinical staging step of 0.5 mm or 3° proposed for this technique [9,27,30]. A mean non-prescribed rotation of 3.32° was observed in the translational movement of tooth 11 and a mean non-prescribed translation of −0.88 mm in the rotational movement of tooth 23. This is in contrast to the fabrication of digital setups, where tooth movements can be performed with a high level of accuracy using a uniform coordinate system [24]. Additionally, the uniform coordinate system allows for a reliable superimposition and precise comparison of tooth movements without the necessity of coordinate transformation, regardless of whether it is applied to a scanned plaster model or an intraoral scan.

The extent to which aligner setups can be clinically realized remains to be investigated, especially with regard to the overall quality of the aligner setup and case-related staging.

## 5. Clinical Implications

Although various aligner manufacturers such as Invisalign^®^ (Align Technology), CA^®^ (Scheu-Dental), or Essix^®^ (Dentsply) rely on the use of computer-aided technology and digital setups, the use of manual setups for the in-house fabrication of aligner models represents a cost-effective alternative method that does not require intraoral scanners and 3D printers [9,30,38,39].

In fact, to date, there are no available accuracy data to oppose the use of manual setups for aligner therapy. One available study even supported the clinical acceptability of manual setups, although only intra-arch and inter-arch measurements were considered [14]. In contrast, the results of the present study suggest that manual setups may not be suitable for the fabrication of aligners with respect to the high variability of results regarding individual tooth movements. Consequently, the manual approach for orthodontic aligner setups should be critically questioned.

## 6. Conclusions

In manual setups performed by dental technicians, tooth movements rarely achieved precise specifications and exhibited unwanted movements in all directions, for both translations and rotations. Based on the wide intra- and inter-technician variability and deviation from the measured values, the manual fabrication of setups should not be favored for aligner therapy.

## Figures and Tables

**Table 1:** Descriptive and inferential statistics of 1 mm translational vestibular (*Z*-axis) movement of tooth 11 and 15° mesial rotation (*Y*-axis) of tooth 23.

Tooth	Type of Movement	Axis of Movement	Expected Movement	Mean (SD)	Median (IQR)	Range	One-Sample Wilcoxon Signed Rank Test	
							Z	P
11	Rotation (°)	X	0	3.32 (2.17)	2.62 [1.77; 4.35]	−0.08 to 10.10	7.056	<0.001
		Y	0	−1.26 (1.51)	−0.91 [−2.15; 0.05]	−5.16 to 1.64	−5.350	<0.001
		Z	0	−1.39 (1.43)	−1.38 [−2.39; −0.81]	−5.63 to 2.57	−5.810	<0.001
	Translation (mm)	X	0	0.20 (0.13)	0.18 [0.09; 0.28]	−0.01 to 0.58	7.043	<0.001
		Y	0	−0.16 (0.26)	−0.13 [−0.33; 0.02]	−1.02 to 0.37	−4.532	<0.001
		Z	1	1.09 (0.42)	1.04 [0.79; 1.39]	0.25 to 2.26	1.434	0.152
23	Rotation (°)	X	0	−1.13 (4.03)	−0.52 [−3.57; 1.70]	−10.38 to 7.58	−1.607	0.108
		Y	15	9.61 (3.46)	9.76 [7.58; 12.19]	2.58 to 18.04	−6.915	<0.001
		Z	0	5.27 (2.99)	4.47 [2.99; 7.02]	−0.32 to 13.21	7.056	<0.001
	Translation (mm)	X	0	−0.26 (0.19)	−0.26 [−0.37; −0.14]	−0.77 to 0.08	−6.749	<0.001
		Y	0	0.16 (0.27)	0.14 [−0.02; 0.33]	−0.34 to 0.77	4.124	<0.001
		Z	0	−0.88 (0.40)	−1.01 [−1.16; −0.75]	−1.47 to 0.02	−7.024	<0.001

Translational movements in the directions x (mesio-distal), y (apical-occlusal), and z (orovestibular) and rotations around the same axes were analyzed. Values are presented as mean, standard deviation (SD), range, median, and interquartile range (IQR). Statistical significances were determined using the one-sample Wilcoxon signed-rank test with Z statistics and *p*-value reported.

## Data Availability

The original data set can be provided upon request.
